# Rolling Egg-Shaped Peritoneal Loose Body (PLB): A Diagnostic Dilemma for Surgeons and Radiologists

**DOI:** 10.7759/cureus.31214

**Published:** 2022-11-07

**Authors:** Nizamuddin Ansari, Parijat Suryavanshi, Gitika N Singh, Shubhajeet Roy, Shreya Verma

**Affiliations:** 1 Department of General Surgery, King George's Medical University, Lucknow, IND; 2 Faculty of Medicine, King George's Medical University, Lucknow, IND

**Keywords:** omental appendices, epiploic appendages, peritoneal mice, peritoneum, giant peritoneal loose body

## Abstract

A man in his mid-50s presented with heaviness in the right lower abdomen for the last three months. Ultrasonography (USG) showed an intra-abdominal mass in the right iliac fossa. Contrast-enhanced CT (CECT) revealed a well-defined circumscribed mass near the ileocaecal junction, with a hypodense center, surrounded by a hyperdense periphery and well-defined capsule. A lower midline laparotomy was performed. Intraoperatively, a white, firm, smooth ball-like mass was found, lying freely in the abdomen. Histopathology revealed fatty tissues surrounded by a calcified shell, which was further surrounded by proteinaceous material. Such peritoneal loose bodies (PLBs) are free masses in the abdomen, with variable sizes. These are asymptomatic, unless they compress any nearby viscera, as was in our case. The objective of this case report is to make surgeons and radiologists aware of this rare entity, which can be a source of confusion in a case of mass in the abdomen.

## Introduction

A peritoneal loose body (PLB) is a rare incidental finding that may lead to diagnostic confusion and undue anxiety due to imaging resemblance to gastrointestinal mesenchymal tumors. This entity is benign and remains largely asymptomatic. Its size may vary, ranging from a few mm to more than 10 cm [1]. A high index of suspicion is required to make a preoperative diagnosis on imaging. Symptoms arise due to these loose bodies compressing the intestines. Here, we present a case report of a man in his mid-50s with vague abdominal pain and a radiological diagnosis of a mesenchymal tumor. The patient was operated on and a PLB was found. The patient has been asymptomatic since then.

## Case presentation

A 55-year-old male who had been complaining of right lower abdominal heaviness for previous three months presented to the surgery outdoor. There were no specific systemic complaints. Clinical examination was normal and no lump could be palpated in the abdomen.

**Figure 1:**
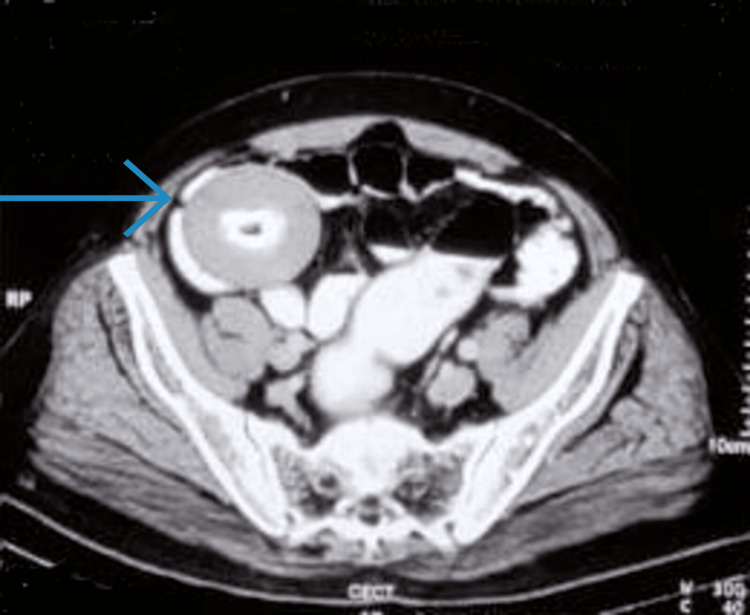
Axial contrast-enhanced CT (CECT) scan of the abdomen with IV and oral contrast demonstrating loose body, which was a hypodense center within a calcified shell surrounded by a coating of hyperdense material (blue arrow)

Initial ultrasonography (USG) of the abdomen revealed an intra-abdominal hypoechoic rounded mass of size about 7 x 6 cm in the right iliac fossa. Contrast-enhanced CT (CECT) scan of the abdomen, performed a month back, showed a well-defined circumscribed mass of size about 7 x 7 x 6.1 cm near the ileocaecal junction, with a hypodense center, surrounded by a hyperdense periphery and a well-defined capsule (Figure 1). The radiologist suspected a tumor arising from the small bowel and a provisional diagnosis of small bowel mesenchymal tumor was made. The patient also had a recent CT scan, which was done before the patient presented to us. It also revealed the same lesion without any significant change in size, though now it lay in the pelvis and slightly compressed the rectum. Hence, it was suspected that it could be a benign small bowel tumor that has rolled into the pelvis along with the bowel loop from where it may have originated.

**Figure 2:**
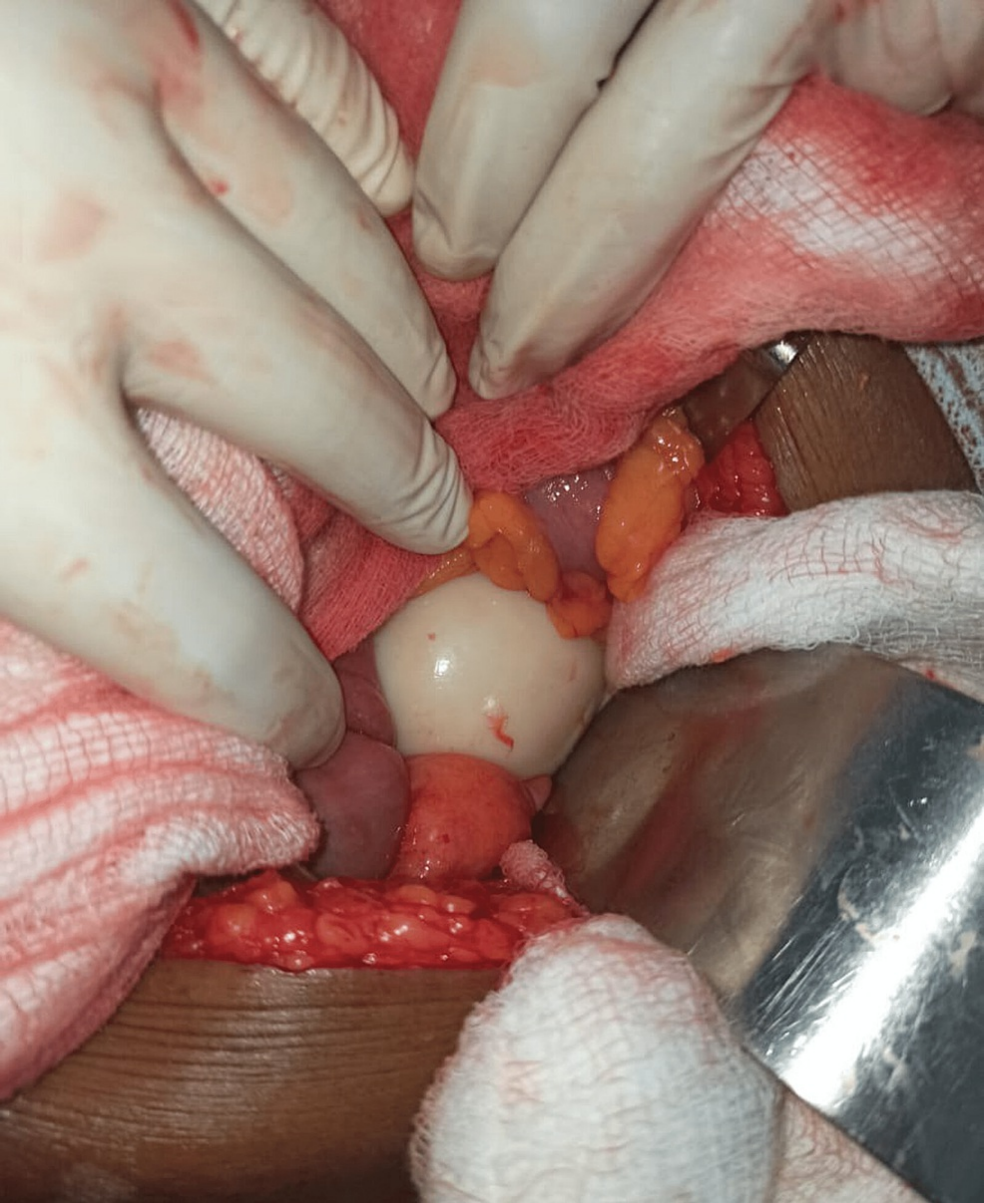
Intraoperative finding of white ball-like mass lying free in the peritoneal cavity

Considering the possibility of intra-abdominal mass originating from bowel, we preoperatively planned for excision of mass with bowel resection and anastomosis and decided to proceed with lower midline laparotomy after taking informed consent from the patient. Intraoperatively, a white firm smooth ball-like mass was found, which was not attached to any viscera or wall, lying freely in the abdomen (Figure 2).

**Figure 3:**
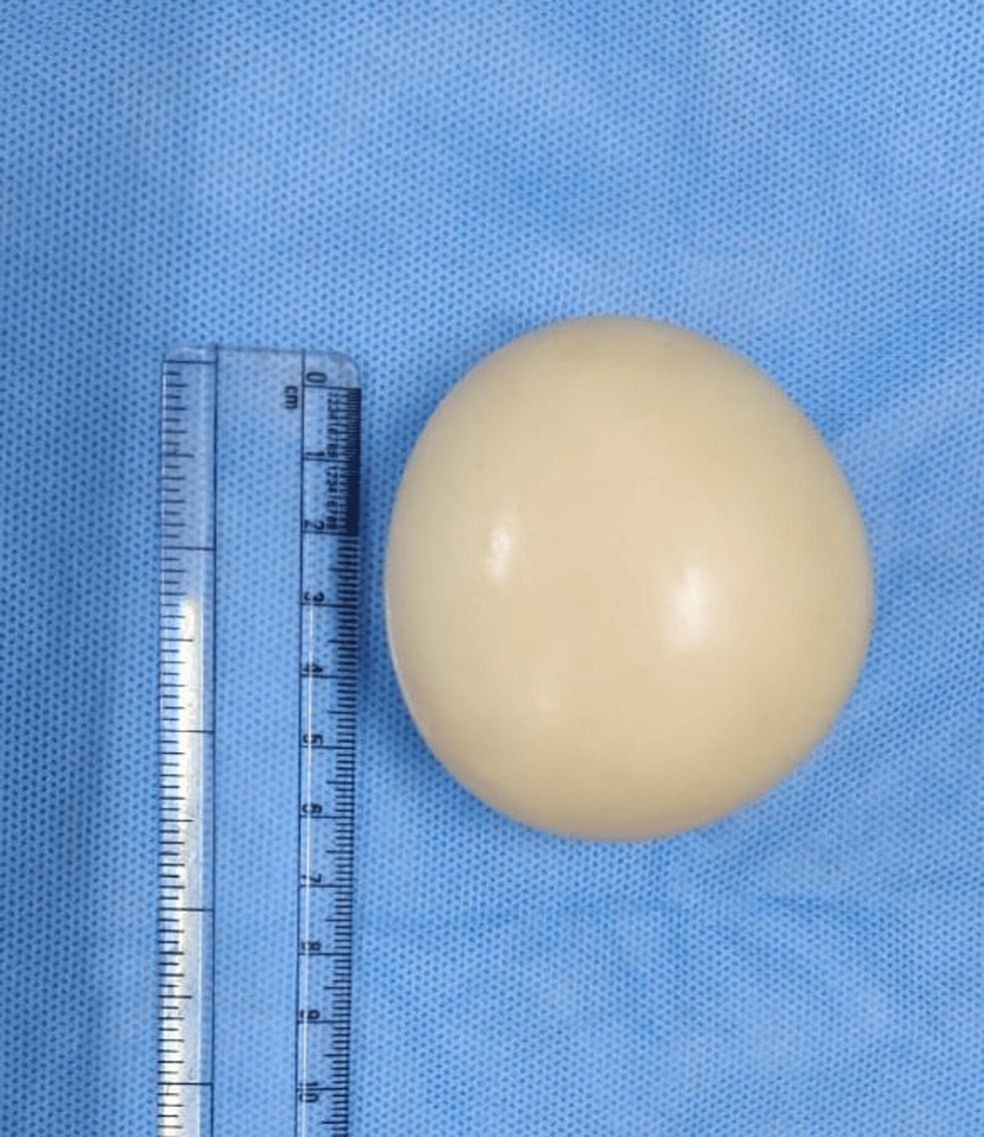
Loose body 7 x 6 cm with smooth glistening surface

The surface of the mass was carefully inspected. It was smooth all around. The rest of the abdomen was inspected and no other abnormal findings could be found. On gross examination, the specimen was 7 x 6 cm, round, ball-like, with a white, smooth, shiny glistening surface, and was firm in consistency (Figure 3).

**Figure 4:**
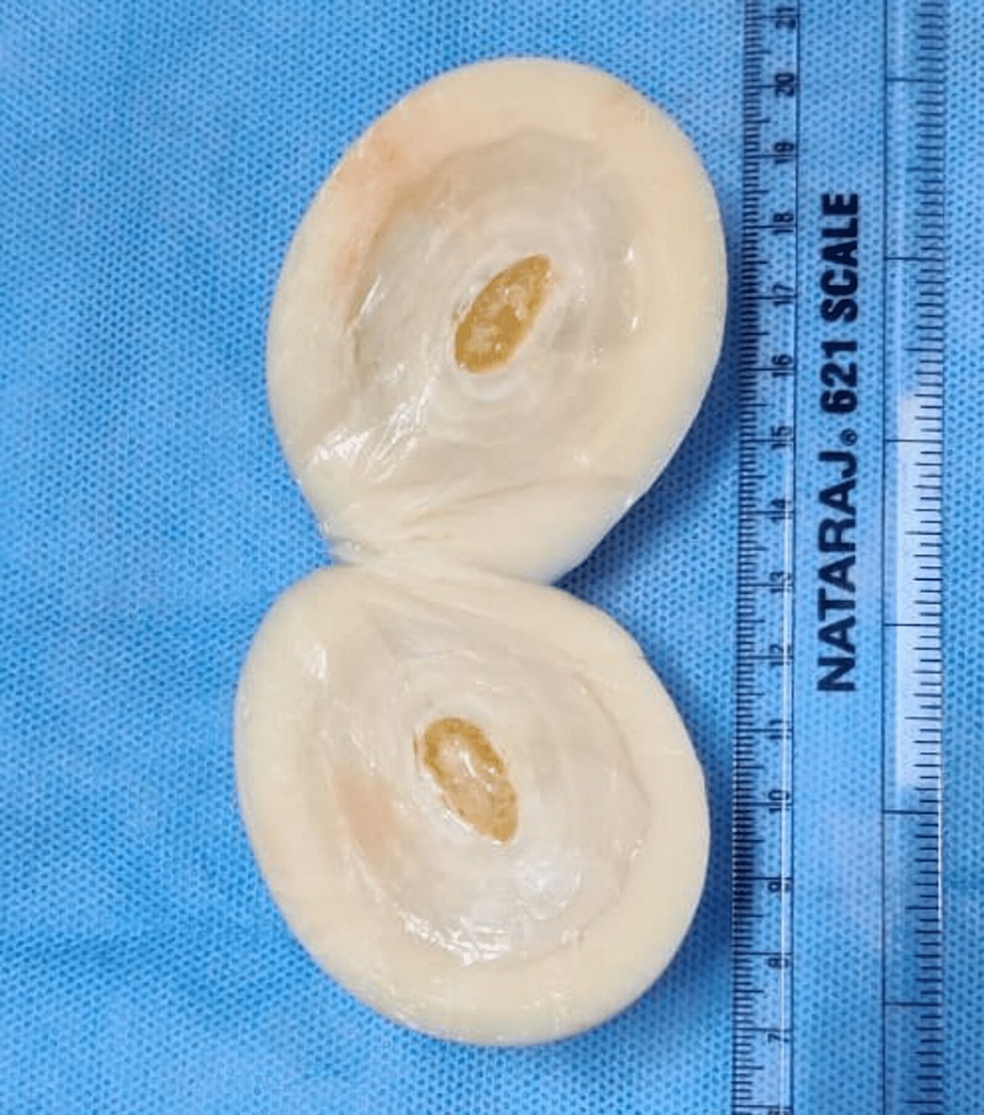
Cut section of the loose body showing fatty center with a calcified shell and proteinaceous coating

The cut section showed an outer area that was rubbery to feel while slicing. In the center, there was an almond-shaped core whose shell was hard and difficult to crack. On cutting the shell, fatty tissue was found inside (Figure 4).

Histopathology revealed fatty tissues surrounded by a calcified shell, which was further surrounded by proteinaceous material. The abdomen was closed without the placement of any drain.

## Discussion

PLBs are free masses in the abdomen, with variable sizes. They are uncommon entities with many cases diagnosed incidentally on being investigated for unrelated complaints. Loose bodies are largely asymptomatic unless they compress any nearby viscera, which usually occurs in large ones, as was in our case [2,3]. Hence, most of them remain undiagnosed, so the exact incidence could not be stated. Most of the available literature states that these bodies were often encountered during autopsy. Various theories have emerged regarding their formation. The most prevalent and accepted theory is that these bodies are formed from shed infarcted appendices epiploic. These epiploic appendages may undergo chronic infarction due to torsion or thrombosis of the central draining vein and then get shed into the peritoneal cavity. Once in the peritoneal cavity, they get bathed in the peritoneal fluid. They undergo saponification, and calcium gets deposited around fatty tissue, forming a shell [4,5]. With time, lying in a pool of protein-rich peritoneal fluid, it acquires a shell of protein from the peritoneal fluid, which keeps on depositing and, hence, increasing the size of the loose body until diagnosed and removed, if at all that happens in one’s lifetime. On CT scan, they appear as a hypodense small center surrounded by a calcified shell and mantle of hyperdense proteinaceous material [6,7]. Treatment could be observation or surgical removal. If the size is large and is causing pressure symptoms, it is often removed to confirm the diagnosis. Laparoscopic removal is also feasible [8].

Differential diagnosis of PLBs include:

1) Leiomyoma uterus: On USG, uncomplicated leiomyomas are usually hypoechoic but can be isoechoic or even hyperechoic compared to normal myometrium. Calcification is seen as an echogenic focus with shadowing. Cystic areas of necrosis or degeneration may be seen. 2) Leiomyoma recto-sigmoid (if lying in the pelvis): On CT scan, it is seen as smooth-surfaced mass with loss of fat planes with recto-sigmoid showing varying degrees of internal necrosis. Moreover, heterogeneous contrast enhancement and dystrophic calcification may also be seen. 3) Gastrointestinal stromal tumor (GIST) of the intestine: Small primary tumors are frequently well marginated and, on unenhanced CT, are seen to have low soft tissue density. On enhanced CT, these tumors, when small, typically show homogeneous enhancement. 4) Foreign body: On CT scan, they show up with irregular margins with adhesion to surrounding structures or scars from previous surgery.

## Conclusions

A PLB can present a difficult diagnostic problem for the surgeon, with confusing findings that may indicate malignancy. This case report aims to alert clinicians about this rare finding of PLBs, which are benign but could be easily confused with well-circumscribed tumors like leiomyoma or GIST. Preoperative diagnosis of these lesions is difficult and a high index of suspicion should be kept in any symptomatic patient with a mobile lesion in the abdomen or a calcified lesion in the pelvis on X-ray. No specific treatment is required in asymptomatic patients. However, if these entities become associated with complications like intestinal obstruction, if there is an abdominal mass of obscure origin, or when the diagnosis is in doubt, then exploration is required. If the size is large and is causing pressure symptoms, it is often removed to confirm the diagnosis. Laparoscopic removal is also feasible. It is also equally important to allay the anxiety of patients

.
